# No effect of double nerve block of the lateral cutaneous nerve and subcostal nerves in total hip arthroplasty: A randomized controlled trial

**DOI:** 10.1080/17453674.2018.1437951

**Published:** 2018-03-01

**Authors:** Johannes L Bron, Jeanette Verhart, Inger N Sierevelt, Dirk De Vries, Hylke J Kingma, Maarten V Rademakers

**Affiliations:** 1 Department of Orthopaedic Surgery, Spaarne Gasthuis Hoofdorp; 2 Spaarne Gasthuis Academy (formerly: Lineaus Institute), Hoofddorp; 3 Department of Anaesthesiology, Spaarne Gasthuis Hoofddorp; 4 Pharmacy Foundation of the Haarlem Hospitals (SAHZ), Haarlem; 5 Currently: Department of Orthopaedic Surgery, Antonius Hospital, Sneek, The Netherlands

## Abstract

**Background and purpose:**

The use of local infiltration anesthesia (LIA) has become one of the cornerstones of rapid recovery protocols in total knee arthroplasty patients during the past decade. In total hip arthroplasty (THR), however, the study results are more variable and LIA has therefore not yet been generally accepted. There is no consensus on which structure should be infiltrated and the cutaneous nerves are generally neglected. Hence, we hypothesized a pain-reducing effect of specifically blocking these nerves.

**Patients and methods:**

We performed a single-center randomized placebo-controlled trial in 162 subjects to evaluate the infiltration of the lateral cutaneous femoral and subcostal nerve with ropivacaine in patients undergoing total hip arthroplasty via a straight lateral approach. The primary endpoint was pain at rest after 24 hours. Patients were followed up to 6 weeks postoperatively.

**Results:**

After correction for multiple testing, no statistically significant differences in pain scores were found between the ropivacaine compared with the placebo group after surgery. In addition, no differences were observed in the use of escape pain medication, complications, and the length of hospital stay.

**Interpretation:**

We found no clinically meaningful differences in pain scores between placebo and ropivacaine patients in the postoperative period after THA performed via a straight lateral approach under spinal anesthesia and a multimodal pain regimen. Moreover, our primary endpoint, pain reduction after 24 hours, was not met. Further research should focus on the composition and volume of the LIA suspension, the optimal localization of the infiltration, and should be evaluated for every surgical approach separately.

While the use of local infiltration anesthesia (LIA) in total knee arthroplasty (TKA) has been embraced by orthopedic surgeons worldwide, the use in total hip arthroplasty (THA) patients has remained a matter of debate (Andersen and Kehlet 2014). LIA in TKA has unequivocally been shown to reduce early postoperative pain with positive effects on mobilization and hospitalization (Andersen et al. 2008a and b, Andersen et al. 2009, Dillon et al. 2012, Gibbs et al. 2012, Tran and Schwarzkopf 2015).

Studies with the use of LIA in THA patients show mixed results (Andersen et al. 2007a, Busch et al. 2010, Andersen et al. 2011, Banerjee and McLean 2011, Lunn et al. 2011, Dobie et al. 2012, Murphy et al. 2012, Rikalainen-Salmi et al. 2012, Kuchalik et al. 2013, Pandazi et al. 2013, Zoric et al. 2014, den Hartog et al. 2015). Although most research consists of well-conducted randomized controlled trials (RCTs), study designs and set-up vary considerably (Wang et al. 2017). Differences are found in the treatment of the control groups, the composition of the LIA suspension, and the endpoints investigated. Moreover, surgical factors like surgical approach and the structures that are actually being infiltrated differ remarkably (Table 1). Our prior observations in THA patients via a straight lateral approach showed that most patients suffer from superficial wound pain instead of deeper groin pain. We therefore hypothesized that blocking the nerves innervating the wound area might result in reduced postoperative pain.

**Table 1.** Overview of the trials with LIA performed in THA patients. Note the remarkable variation in the LIA suspension, localization of infiltration, surgical approach, and endpoint studied

Study	N	Set up	Control	Approach**^a^**	Suspension**^b^**	Infiltration **^c^**	Results
Bianconi et al. 2003	?	RCT	NaCl	PL	R	SC	Lower VAS first 72h
Andersen et al. 2007a/b	80	RCT	epidural	PL	R/K/A	WI + IA	2 days earlier discharge
Andersen et al. 2007a/b	40	RCT	NaCl	PL	R/K/A	WI	Less pain 2 weeks, better function after 1 week
Busch et al. 2010	64	RCT	–	SL	R/K/A	C + SC	Less pain/medication use first 24 h
Lunn et al. 2011	120	RCT	NaCl	PL	R/A	C + M + SC	No differences in pain score
Andersen et al. 2011	12 ^d^	RCT	NaCl	PL	R/A	WI	No differences in pain score and length of stay
Banerjee&McLean 2011	204	retro	–	?	R/K/A	WI	Faster mobilisation and discharge
Murphy et al. 2012	91	RCT	NaCl	PL	bupi	C + M + SC	Les opioid consumption first 12 h
Dobie et al. 2012	96	RCT	–	PL	Bupi/A	LC + C+M + SC	No differences pain/mobilisation
Rikalaine et al. 2012	60	RCT	–	?	B/K/A	C + M + SC	No difference VAS, less urinary retention, faster mobilisation
Kuchalik et al. 2013	80	RCT	epidural	?	R/K/A	C + M + SC	Less pain during mobilisation less escape medication
Pandazi et al. 2013	63	RCT	PCA	?	R/K/A	C + M	Less pain and opioid consumption first 24h
Zoric et al. 2014	60	RCT	NaCl	PL	R	C + M + SC	No differences opioid consumption/mobilisation
den Hartog et al. 2015	75	RCT	NaCl	A	R/A	C + M + SC	No differences

**^a^** Approach: PL = posterolateral, SL = straight lateral, A = anterior

**^b^** Suspension: R = ropivacaine, K = ketorolac, A = adrenalin

**^c^** Infiltration: SC = subcutaneous, WI = wound infiltration, IA = intraarticular, C = capsule, M = muscle, LC = lateral cutaneous nerve

**^d^** bilateral

This study is a single-center, randomized placebo-controlled trial in patients undergoing THA via straight lateral approach by infiltrating the nerves innervating the area of the incision—the lateral cutaneous femoral nerve and the subcostal nerve—with ropivacaine compared with placebo (NaCl 0.9%). All patient received the ropivacaine or placebo in addition to multi-modal pain management in a fast-track recovery protocol. The primary endpoint was pain at rest 24 hours after surgery.

## Patients and methods

## Patients

After written informed consent had been obtained, 162 patients with primary osteoarthritis undergoing elective THA at the Department of Orthopedic Surgery, Spaarne Gasthuis Hospital location Hoofddorp, from August 2014 to September 2016 were enrolled in this randomized trial. Exclusion criteria included known allergy to ropivacaine, general anesthesia, opioid dependency, malignancies, co-morbidities compromising pain perception (e.g., neurologic of psychiatric disorders), ASA grades 3–4. In addition, patients suffering from peroperative complications interfering with postoperative pain and perception or mobilization were excluded. Preoperatively, patient demographics, visual analogue pain scores (VAS) in rest and during mobilization, as well as the Hip disability and Osteoarthritis Outcome Score (HOOS), Short form Survey (SF-12) and Oxford Hip Score (OHS) questionnaires were documented.

### Methods

All patients were admitted on the day of surgery. A standardized pain protocol was used. 2 hours preoperatively patients received 1000 mg acetaminophen, 15 mg meloxicam, and 600 mg gabapentin. All surgeries were performed under spinal anesthesia, using low-dose bupivacaine. Postoperative patients received 1000 mg acetaminophen every 6 hours, 15 mg meloxicam once daily both at least until discharge, and 600 mg gabapentin 3 times a day for a period of 5 days. When the protocol medication was insufficient to control pain, escape medication consisting of Oramorph oral solution (morphine sulfate) or Dipidolor (piritramide) intramuscular (both 5–10 mg) was available and its usage documented.

On the day of surgery, blinded numbered 50 cc syringes containing either ropivacaine (ropivacaine HCl 7.5 mg/mL injection fluid; Fresenius Kabi, Zeist, The Netherlands) or placebo (0.9% NaCl) were provided by the department of pharmacy, which used a block randomization scheme to control group volume. Surgeons, patients, nurses, and investigators were all blinded for treatment. Standard hip arthroplasty surgeries (uncemented Zweymuller steel, Durasul cup (Zimmer Orthopaedics)) were performed by 6 different orthopedic surgeons or coupled residents via a straight lateral approach according to Hardinge. Prior to the surgeries an instruction video was shown to all surgeons on multiple occasions. The video shows exactly how to infiltrate with the highest chance of blocking the targeted nerves. In addition, a single physician assistant was instructed to control the infiltration and was present at the theatre at all surgeries. At the end of the surgeries before wound closure, 0.5 mL/kg body weight of the syringes was infiltrated at 2 different locations: (1) half of the volume was infiltrated anterior to the superior iliac spine, where the LCFN passes below the inguinal ligament; (2) the other half of the volume was infiltrated in the tissues of the upper part of the wound extending from the anterior iliac spine to the iliac crest just cranial of the wound to anaesthetize all sensible end nerves of the subcostal nerves.

Postoperatively, VAS at rest, escape medication, and complications every 6 hours on the first day and every 8 hours on the second day were registered. Nausea, vomiting, and urinary retention were also documented. The primary endpoint of the study was the VAS at rest 24 hours postoperatively. Discharge criteria included stable vital signs, the ability to walk with crutches, to walk the stairs, and VAS <4. After discharge, patients were seen at the outpatient clinic 6 weeks postoperatively and the HOOS, SF-12, and OHS questionnaires as well as complications were documented. After the last patient was seen in the outpatient clinic the randomization key was provided to the investigators by the pharmacy department.

### Statistics

Intention to treat analyses were performed by use of IBM SPSS Statistics for Windows version 24 (IBM Corp, Armonk, NY, USA). All variables are summarized according to their distribution by use of means with standard deviations (SD) or frequencies with accompanying percentages. Patient characteristics and baseline variables were compared between treatment groups using Student’s t-tests and chi-squared tests (or Fisher’s exact tests) for continuous and categorical variables respectively. The effect of the intervention on patient-reported outcome measures (NRS pain, HOOS, OHS, and SF-12) was analyzed by use of univariate and multivariate regression analysis to correct for potential confounders (age, gender, BMI) at primary (24 hours) and secondary endpoints, and mean between group difference with 95% confidence intervals were calculated at each follow-up moment. Additionally, escape medication usage and complications were compared by use of chi-squared tests (or Fisher’s exact tests). For the main analysis (NRS pain at 24 hours) a p-value of ≤0.05 was considered statistically significant. Holm’s procedure was used to correct for multiple testing for secondary outcomes. Sensitivity analysis, using multiple imputation, was performed for all cases to assess presence of bias. 10 fully observed datasets were imputed with complete data and were analyzed to conform with the previously stated protocol, and results were aggregated.

### Ethics, registration, funding, and potential conflicts of interest

The randomized double-blind placebo-controlled study was approved by the local ethics committee (protocol no. M013-034). Written informed consent was obtained from all participants. The study was registered at EudraCT registry (2013-004031-71) and Dutch CCMO registry (NL45740.094.13). No competing interest declared. No funding was received for this study.

## Results

**Figure 1.**
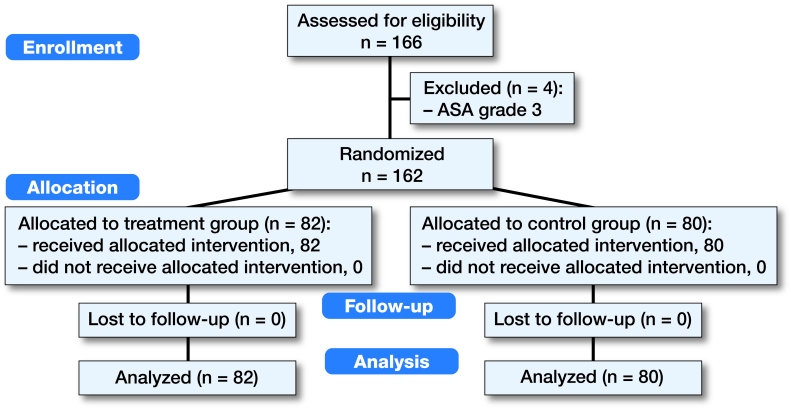
Flow chart of the study.

**Figure 2.**
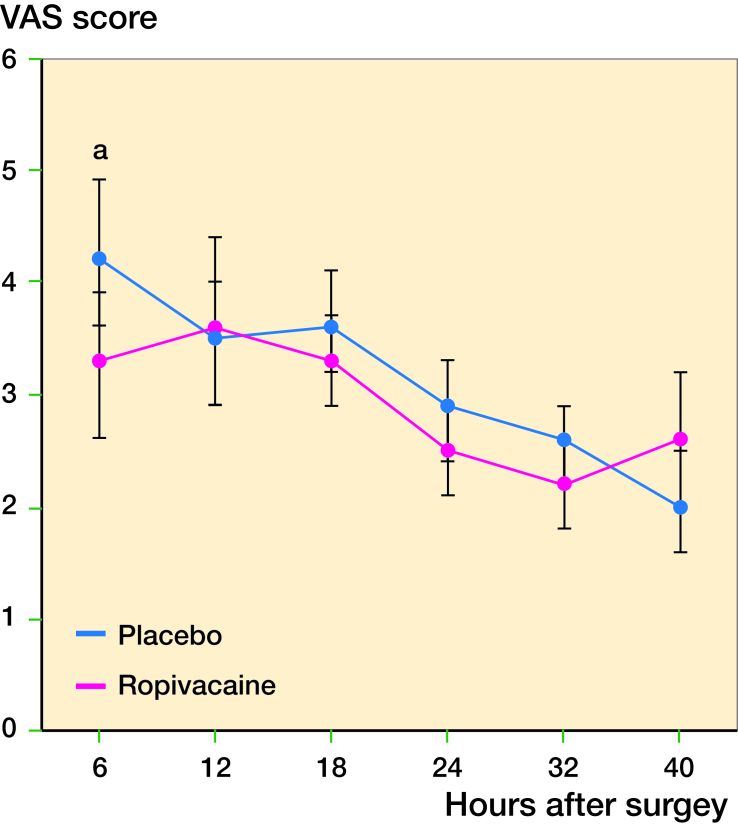
Pain scores of both groups decline in time postoperatively. **^a^** At the first time point the LIA group has a statistically significant lower pain score compared with the placebo group.

166 patients were randomized into 2 groups (Figure 1). 4 of the subjects were excluded due to their ASA (ASA III) classification, which was a pre-defined exclusion criterion. Of the remainder 80 were enrolled in the placebo group and 82 in the ropivacaine group. No subjects were lost to follow-up. Baseline characteristics and patient demographics are given in Table 2. The mean age of the subjects was 66 years in both groups with a female predominance. The results of the VAS measurements are shown in Table 3 and Figure 2. After 6 hours there was a statistically lower VAS at rest in the study group compared with the control (p = 0.04). At later time points, this difference ceased (Figure 2). When using Holm’s procedure to correct for multiple comparisons, however, the significance after 6 hours was lost (significance level according to Holm’s procedure <0.008). There is no statistically significant difference in the use of escape pain medication between the two groups. In addition, no differences are observed in the HOOS, OHS, and SF-12 questionnaires. The occurrence of nausea, vomiting, and urinary retention was equally distributed between the two groups (data not shown). The presence of adverse events was also similar between the two groups, with none of the events related to the infiltration of ropivacaine. The 3 serious adverse events included one subject with postoperative hemorrhage requiring surgical evacuation (ropivacaine group), cardiac ischemia requiring coronary bypass surgery (ropivacaine group), and a subject with early periprosthetic joint infection (placebo group). Finally, the length of stay was not statistically different between the groups (data not shown). Sensitivity analysis revealed similar outcomes, except after 6 hours at which point the difference was not statistically significant anymore. 

**Table 2.** Overview of patient demographics and baseline characteristics in both groups

	Placebo (n = 80)	Ropivacaine (n = 82)
Sex, n (%)		
Male	32 (40)	36 (44)
Female	48 (60)	46 (56)
Age, mean (SD)	66 (9.4)	67 (8.7)
BMI, mean (SD)	28 (4.9)	28 (5.0)
ASA, n (%)		
ASA 1	22 (28)	24 (29)
ASA 2	58 (72)	58 (71)
HOOS, mean (SD)		
Symptoms	45 (16)	40 (16)
Pain	46 (17)	45 (18)
ADL	46 (16)	44 (15)
Sport/rec	25 (18)	31 (24)
QOL	30 (12)	27 (14)
OHS, mean (SD)	23 (7.4)	24 (7.6)
SF12, mean (SD)		
PCS	42 (5.4)	40 (6.3)
MCS	45 (5.7)	46 (5.2)

No statistically significant differences are observed.

**Table 3.** Overview of results of pain scores and questionnaires in both groups. Values are mean (95% CI)

			Between group difference		
	Placebo	Ropivacaine	Unadjusted	Adjusted**^a^**	p-value
NRS pain at rest					
6 hours	4.2 (3.6–4.9)	3.3 (2.6–3.9)	0.9 (0.1 to 1.7)	0.9 (0.04 to 1.7)	0.04
12 hours	3.5 (2.9–4.0)	3.6 (2.9–4.4)	−0.1 (-1.1 to 0.8)	−0.2 (-1.1 to 0.8)	0.8
18 hours	3.6 (3.2–4.1)	3.3 (2.9–3.7)	0.3 (-0.3 to 0.9)	0.3 (-0.3 to 0.9)	0.3
24 hours	2.9 (2.4–3.3)	2.5 (2.1–2.9)	0.4 (-0.2 to 0.9)	0.4 (-0.2 to 1.0)	0.2
32 hours	2.6 (2.2–2.9)	2.2 (1.8–2.6)	0.4 (-0.1 to 0.8)	0.4 (-0.1 to 0.8)	0.2
40 hours	2.0 (1.6–2.5)	2.6 (2.0–3.2)	−0.5 (-1.3 to 0.2)	−0.6 (-1.3 to 0.2)	0.1
HOOS (at 6 weeks)	(n = 53)	(n = 57)			
Symptoms	74 (70–79)	74 (70–79)	−0.2 (-6.4 to 6.1)	−0.2 (-6.0 to 6.5)	0.9
Pain	83 (79–87)	82 (78–86)	0.7 (-5.2 to 6.6)	0.5 (-5.5 to 6.5)	0.9
ADL	77 (73–82)	78 (74–82)	−0.7 (-6.8 to 5.4)	−0.8 (-7.0 to 5.4)	0.8
Sports/Rec	55 (48–62)	58 (50–65)	−2.5 (-12.6 to 7.7)	−2.3 (-12.7 to 8.0)	0.7
QOL	57 (51–62)	61 (55–67)	−4.3 (-12.4 to 3.8)	−4.3 (-12.5 to 3.8)	0.3
OHS (at 6 weeks)	13 (11–15)	12 (10–14)	1.1 (-1.7 to3.8)	1.1 (-1.7–3.9)	0.4
SF12 (at 6 weeks)					
PCS	45 (43–47)	45 (43–48)	−0.6 (-3.7 to 2.6)	−0.7 (-3.9 to 2.5)	0.7
MCS	45 (43–46)	44 (42–46)	0.6 (-2.3 to 3.5)	0.6 (-2.4 to 3.5)	0.7

**^a^** Between-group differences adjusted for age, gender, and BMI.

Only at T = 6 hours is the pain score in the LIA group significantly lower compared with the placebo group with a p-value of 0.04. However, note that the p-value required for significance is < p = 0.008 after Holm’s procedure.

## Discussion

We found that a double nerve block of the lateral cutaneous nerve and subcostal nerves does not result in significant pain relief in the postoperative period. No differences in complications or other adverse events were observed. Moreover, the pre-determined primary endpoint of the current trial, a lower pain score after 24 hours, was not met. Although initially a significant reduction of pain scores after 6 hours was found, the difference was lost after using a multiple comparisons procedure to control for the inflation of the type 1 error rate. And even when significance had remained, it was not clinically relevant, since the VAS was only 0.9 point in favor of the LIA group; it has been suggested that differences in VAS become clinically meaningful when exceeding 1.3 points (Gallagher et al. 2001). A well-known limitation of the VAS measurements is the ceiling effect that becomes more significant at later time points due to the low values that approach the values of healthy subjects.

Postoperative pain is a complex clinical condition that is still not fully understood. One of the main causes is the local tissue damage due to the surgical approach. This may be the main reason why LIA has reliable outcomes in TKA patients in whom the surgical approach hardly differs between trials. In THA on the other hand, there is a highly variable surgical preference to approaching the hip, which is reflected by the different approaches used in LIA studies (see Table 1). It is important to mention that local infiltration is performed as part of an effective multimodal pain regimen consisting of acetaminophen, meloxicam and gabapentin. The latter especially is not always well tolerated and may result in early postoperative dizziness and more effective local infiltration might therefore allow a reduction of these medications.

We used a straight lateral approach, which was only used in 2 prior studies (Bianconi et al. 2003, Busch et al. 2010). Both studies showed a reduction in postoperative pain by the use of LIA. However, further comparison with our trial is controversial since the groups were much smaller compared with our group and the tissues that were infiltrated differed. Both RCTs used LIA to infiltrate primarily the subcutaneous tissues and Busch et al. also targeted the capsule tissue. Our aim was to show whether the infiltration of the nerves supplying the skin around the incision area is of additional value as a part of traditional LIA. The reason for this type of infiltration is that in our prior observations most patient suffer from superficial wound pain after a THA performed by a straight lateral approach; the groin pain after surgery is mostly very mild. Only a single study infiltrated the lateral cutaneous femoral nerve, but the surgeries in that study were actually performed via a posterolateral approach (Dobie et al. 2012). In that study also, no differences were found in pain and mobilization in the LIA compared with a not further specified control group. The study, however, additionally infiltrated the surgical tissues and had a much smaller sample size than our study.

One of the possible limitations of our study is that the suspension contained only ropivacaine. Earlier studies in TKA showed superior results with the addition of an anti-inflammatory agent such as ketorolac (Andersen et al. 2013). Also, studies that used ketorolac in the suspension in THA generally show more favorable results (Andersen et al. 2007a, Andersen et al. 2007b, Busch et al. 2010, Banerjee and McLean, 2011, Kuchalik et al. 2013). Our aim, however, was to block the nerves supplying the surgical area in order to evaluate specifically the pain contribution from these 2 nerves and we did not directly infiltrate the damaged tissues in the surgical field. It might therefore be questioned whether the addition of ketorolac would have been more effective. Moreover, the addition of ketorolac to the LIA suspension can be debated by the absence of NSAID receptors in the tissues around the hip and the agents may act primarily by a general instead of a local effect. We did administer an NSAID to all patients orally postoperatively. The only trial studying the infiltration of the cutaneous femoral nerve as part of LIA used bupivacaine (and adrenalin (epinephrine)) (Dobie et al. 2012).

Our primary endpoint was pain reduction after 24 hours. It might be questioned whether ropivacaine is still working at that time point, in view of its half-life of 2–6 hours. However, we surmised if pain was reduced early postoperatively this would have resulted in earlier effective mobilization and thereby a longer reduction of pain and earlier discharge. Obviously, choosing an earlier endpoint in the current study would not have resulted in another conclusion since no differences were observed at all. The maximum follow-up was 6 weeks, since we did not expect any differences in the long term. Few studies have looked at the long-term effects of LIA in arthroplasty patients. Interestingly, a recent study showed that LIA in hip arthroplasty patients reduced postsurgical pain after 1 year (Wylde et al. 2015). Because of these findings, they also showed that the LIA administration is more cost effective in hip compared with knee arthroplasty patients (Marques et al. 2015). Since in our study all differences have ceased after 6 hours postoperatively, the interesting findings in these trials seem incompatible with our results.

In summary, we show that a double nerve block of the lateral cutaneous nerve and subcostal nerves does not result in a clinically relevant reduction of postoperative pain in THA patients with surgery performed by a straight lateral approach under spinal anesthesia. Further research should focus on the composition and volume of the LIA suspension, the optimal localization of the infiltration, and should be evaluated for every surgical approach separately.

The authors would like to thank all the patients for their participation in the study. In addition, the authors are grateful to Paul Spruijt, José de Droog and the orthopedic surgeons performing the surgeries as well as the people involved in the outpatient clinic, operating theater, and clinical department.

JLB and MV designed and coordinated the study, JV was involved in data collection, INS performed the statistical analysis, DdV wrote the pain protocol, HJK performed the randomization, JLB drafted the manuscript and MV revised the manuscript.

*Acta* thanks Henrik Husted and Johan Rader for help with peer review of this study.

## References

[C1] Andersen L O, Kehlet H. Analgesic efficacy of local infiltration analgesia in hip and knee arthroplasty: a systematic review. Br J Anaesth 2014; 113: 360–74. 24939863 10.1093/bja/aeu155

[C2] Andersen K, Pfeiffer-Jensen M, Haraldsted V, Søballe Kjeld. Reduced hospital stay and narcotic consumption, and improved mobilization with local and intraarticular infiltration after hip arthroplasty. Acta Orthop 2007a; 78: 180. 17464604 10.1080/17453670710013654

[C3] Andersen L J, Poulsen T, Krogh B, Nielsen T. Postoperative analgesia in total hip arthroplasty: a randomized double-blinded, placebo-controlled study on peroperative and postoperative ropivacaine, ketorolac, and adrenaline wound infiltration. Acta Orthop 2007b; 78: 187–92. 17464605 10.1080/17453670710013663

[C4] Andersen L Ø, Husted H, Otte K S, Kristensen B B, Kehlet H. High-volume infiltration analgesia in total knee arthroplasty: a randomized, double-blind, placebo-controlled trial. Acta Anaesthesiol Scand 2008a; 52: 1331–5. 19025523 10.1111/j.1399-6576.2008.01777.x

[C5] Andersen L Ø, Husted H, Otte K S, Kristensen B B, Kehlet H. A compression bandage improves local infiltration analgesia in total knee arthroplasty. Acta Orthop 2008b; 79: 806–11. 19085499 10.1080/17453670810016894

[C6] Andersen L Ø, Gaarn-Larsen L, Kristensen B B, Husted H, Otte K S, Kehlet H. Subacute pain and function after fast-track hip and knee arthroplasty. Anaesthesia 2009; 64: 508–13. 19413820 10.1111/j.1365-2044.2008.05831.x

[C7] Andersen L Ø, Otte K S, Husted H, Gaarn-Larsen L, Kristensen B, Kehlet H. High-volume infiltration analgesia in bilateral hip arthroplasty: a randomized, double-blind placebo-controlled trial. Acta Orthop 2011; 82: 423–6. 21751861 10.3109/17453674.2011.596063 PMC3237031

[C8] Andersen K V, Nikolajsen L, Haraldsted V, Odgaard A, Søballe K. Local infiltration analgesia for total knee arthroplasty: should ketorolac be added? Br J Anaesth 2013; 111: 242–8. 23514638 10.1093/bja/aet030

[C9] Banerjee P, McLean C. The efficacy of multimodal high-volume wound infiltration in primary total hip replacement. Orthopedics 2011; 34: e522–9. 21902151 10.3928/01477447-20110714-11

[C10] Bianconi M, Ferraro L, Traina G C, Zanoli G, Antonelli T, Guberti A, et al Pharmacokinetics and efficacy of ropivacaine continuous wound instillation after joint replacement surgery. Br J Anaesth 2003; 91: 830–5. 14633754 10.1093/bja/aeg277

[C11] Busch C A, Whitehouse M R, Shore B J, MacDonald S J, McCalden R W, Bourne R B. The efficacy of periarticular multimodal drug infiltration in total hip arthroplasty. Clin Orthop Relat Res 2010; 468: 2152–9. 20020333 10.1007/s11999-009-1198-7 PMC2895844

[C12] Dillon J P, Brennan L, Mitchell D. Local infiltration analgesia in hip and knee arthroplasty: an emerging technique. Acta Orthop Belg 2012; 78: 158–63. 22696983

[C13] Dobie I, Bennett D, Spence D J, Murray J M, Beverland D E. Periarticular local anesthesia does not improve pain or mobility after THA. Clin Orthop Relat Res 2012; 470: 1958–65. 22270468 10.1007/s11999-012-2241-7 PMC3369082

[C14] Gallagher E J, Liebman M, Bijur P E. Prospective validation of clinically important changes in pain severity measured on a visual analog scale. Ann Emerg Med 2001; 38: 633–8. 11719741 10.1067/mem.2001.118863

[C15] Gibbs D M R, Green T P, Esler C N. The local infiltration of analgesia following total knee replacement: a review of current literature. J Bone Joint Surg Br 2012; 94: 1154–9. 22933484 10.1302/0301-620X.94B9.28611

[C16] den Hartog Y M, Mathijssen N M C, van Dasselaar N T, Langendijk P N J, Vehmeijer S B W. No effect of the infiltration of local anaesthetic for total hip arthroplasty using an anterior approach: a randomised placebo controlled trial. Bone Joint J 2015; 97-B: 734–40. 26033051 10.1302/0301-620X.97B6.35343

[C17] Kuchalik J, Granath B, Ljunggren A, Magnuson A, Lundin A, Gupta A. Postoperative pain relief after total hip arthroplasty: a randomized, double-blind comparison between intrathecal morphine and local infiltration analgesia. Br J Anaesth 2013; 111: 793–9. 23872462 10.1093/bja/aet248

[C18] Lunn T H, Husted H, Solgaard S, Kristensen B B, Otte K S, Kjersgaard A G, et al Intraoperative local infiltration analgesia for early analgesia after total hip arthroplasty: a randomized, double-blind, placebo-controlled trial. Reg Anesth Pain Med 2011; 36: 424–9. 21610559 10.1097/AAP.0b013e3182186866

[C19] Marques E M, Blom A W, Lenguerrand E, Wylde V, Noble S M. Local anaesthetic wound infiltration in addition to standard anaesthetic regimen in total hip and knee replacement: long-term cost-effectiveness analyses alongside the APEX randomised controlled trials. BMC Med 2015; 13: 151. 26116078 10.1186/s12916-015-0389-1 PMC4496938

[C20] Murphy T P, Byrne D P, Curtin P, Baker J F, Mulhall K J. Can a periarticular levobupivacaine injection reduce postoperative opiate consumption during primary hip arthroplasty? Clin Orthop Relat Res 2012; 470: 1151–7. 21960156 10.1007/s11999-011-2108-3 PMC3293978

[C21] Pandazi A, Kanellopoulos I, Kalimeris K, Batistaki C, Nikolakopoulos N, Matsota P, et al Periarticular infiltration for pain relief after total hip arthroplasty: a comparison with epidural and PCA analgesia. Arch Orthop Trauma Surg 2013; 133: 1607–12. 24036613 10.1007/s00402-013-1849-8

[C22] Rikalainen-Salmi R, Förster J G, Mäkelä K, Virolainen P, Leino K A, Pitkänen M T, et al Local infiltration analgesia with levobupivacaine compared with intrathecal morphine in total hip arthroplasty patients. Acta Anaesthesiol Scand 2012; 56: 695–705. 22404241 10.1111/j.1399-6576.2012.02667.x

[C23] Tran J, Schwarzkopf R. Local infiltration anesthesia with steroids in total knee arthroplasty: a systematic review of randomized control trials. J Orthop 2015; 12: S44–S50. 26719628 10.1016/j.jor.2015.01.017 PMC4674543

[C24] Wang Y, Gao F, Sun W, Wang B, Guo W, Li Z. The efficacy of periarticular drug infiltration for postoperative pain after total hip arthroplasty: a systematic review and meta-analysis. Medicine (Baltimore) 2017; 96(12): e6401. 28328836 10.1097/MD.0000000000006401 PMC5371473

[C25] Wylde V, Lenguerrand E, Gooberman-Hill R, Beswick A D, Marques E, Noble S, et al Effect of local anaesthetic infiltration on chronic postsurgical pain after total hip and knee replacement: the APEX randomised controlled trials. Pain 2015; 156: 1161–70. 25659070 10.1097/j.pain.0000000000000114 PMC4450871

[C26] Zoric L, Cuvillon P, Alonso S, Demattei C, Vialles N, Asencio G, et al Single-shot intraoperative local anaesthetic infiltration does not reduce morphine consumption after total hip arthroplasty: a double-blinded placebo-controlled randomized study. Br J Anaesth 2014; 112: 722–8. 24431385 10.1093/bja/aet439

